# Immune checkpoint inhibition enhances memory T-cell responses to CEF(T), SARS-CoV-2 spike proteins and ChAdOx1 nCoV-19

**DOI:** 10.1080/21645515.2026.2742035

**Published:** 2026-10-06

**Authors:** Sophie Grice, Joshua Gardner, Paul Thomson, Luisa Hering, Lonnie MacDonald, Stacey Collins, Lance Turtle, Daniel F. Carr, Anna Olsson-Brown, Sean Hammond, Dean J. Naisbitt

**Affiliations:** a Centre for Drug Safety Science, Department of Pharmacology and Therapeutics, University of Liverpool, Liverpool, UK; b Institute of Infection and Global Health, University of Liverpool, Liverpool, UK; c Sussex Cancer Centre, University Hospitals Sussex, Brighton, UK; d ApconiX, Alderley Edge, UK

**Keywords:** Immune checkpoint inhibitor, immunotherapy, vaccines, COVID-19, T-cells, cytokines

## Abstract

An extensive body of clinical and experimental evidence demonstrates that immune checkpoint blockade can invigorate recall immune responses. This phenomenon has previously been highlighted using *ex vivo* (healthy donor) and *in vivo* (immunotherapy patients) models of immune checkpoint inhibitor (ICI) administration that demonstrate an enhancement of the T-cell response to concomitant small-molecule drugs. However, the immunological and clinical impact of ICI therapy pre-ceding vaccination, where robust antigen-specific immunogenicity is essential, remains poorly defined. In this study, we utilized an *ex vivo* assay to evaluate lymphocyte recall responses within peripheral blood mononuclear cells (PBMC) derived from healthy donors and patients pre- and post-immunotherapy following exposure to SARS-CoV-2 derived peptides and ChAdOx1 nCoV-19. In line with the anticipated effects of the primary pharmacology of immune checkpoint inhibitors, we outline an acute increase in proliferation and cytokine and cytolytic molecule secretion following immune checkpoint blockade in response to CEF(T), wild-type SARS-CoV-2 spike pools 1 and 2, and ChAdOx1 nCoV-19. Future studies are needed to evaluate the clinical correlates of the veracity of such responses with protection and incidence of adverse events.

## Introduction

Therapeutic monoclonal antibodies (mAbs) targeting the PD-1 and CTLA-4 pathways are stalwarts of the immune checkpoint class of immuno-oncology agents. The mechanistic principle of these agents is to enhance *de novo* and existing lymphocyte responses to cancers.^1,2^ By the same token, previous work has illustrated that this immunomodulation extends to other antigens and that ICI mediated enhancement of recall responses can be evaluated *in vitro* and *ex vivo*.^3^ This has translational implications for the responses observed to vaccines administered in patients undergoing ICI therapy. Commonly, combination therapy with ICIs is pursued for tumor associated/neoantigenic therapeutic vaccine approaches as a rational strategy to enhance potential efficacy.^4^ A parallel consideration is the impact that ICIs have on the responses engendered by prophylactic vaccines for infectious diseases. A key question for the field which has been reignited by recent data is whether enhanced efficacy^5,6^ and/or potential adverse effects^7^ related to enhanced T-cell responses may occur due to immunological interactions between ICIs and vaccination.

Herein, an evaluation of ICI modulated T-cell responses was explored via the use of a combination of *in vitro* and *ex vivo* experiments. Assays utilizing *in vitro* checkpoint inhibition were conducted on healthy donor derived PBMC challenged with CEF and CEFT peptide pools (containing HLA class I and II restricted epitopes for cytomegalovirus (HCMV), Epstein-Barr Virus (EBV), influenza virus and tetanus toxin). Similar studies were also conducted for SARS-CoV-2 peptide pool responses arising in PBMC sampled longitudinally from healthy volunteers at various stages of vaccination course. Finally, the translational relevance of these responses to the *in vivo* scenario was evaluated through the study *ex vivo* responses to SARS-CoV-2 peptides within patients with cancer undergoing active PD-1/PD-L1 targeted immunotherapy. The outputs of this study are mechanistic in nature and lay the groundwork for future translational prospective studies.

The SARS-CoV-2 virus is composed of a spike glycoprotein, membrane protein, nucleoprotein, genomic RNA and envelope small membrane protein.^8^ The spike glycoprotein is composed of two subunits S1 and S2 and is essential for receptor recognition (ACE2 receptor) and the process of cell membrane fusion.^8^ In this study, we aimed to establish whether recall responses to SARS-CoV-2-associated antigens (S1 and S2 overlapping peptide pools and ChAdOx1 nCoV-19, an adenoviral-based COVID-19 vaccine encoding the full-length, wild type SARS-CoV-2 spike glycoprotein) were affected by immune checkpoint blockade.

## Methods

### Materials and cell culture medium

Peripheral blood mononuclear cells (PBMC) were cultured in “R9” medium; RPMI 1640 (Sigma Aldrich, St. Louis, MO, USA) supplemented with heat inactivated human blood type AB serum (5%; Innovative Research, Novi, MI, USA), L-glutamine (2 mM; Sigma Aldrich), HEPES (4-(2-hydroxyethyl)-1-piperazineethanesulfonic acid (25 mM; Sigma Aldrich), transferrin (25 μg/ml; Sigma Aldrich) and penicillin-streptomycin solution (penicillin 100 IU/mL; streptomycin 100 μg/mL; Sigma Aldrich).

### Healthy donor and patient samples

Isolated PBMCs from vaccinated SARS-CoV-2 convalescent donors were obtained from the University of Liverpool Acute Virus Immunity Study (AVIS, ethics approval ref 16/NW/0170). Healthy volunteers assessed for recall responses to SARS-CoV-2 spike pools 1 and 2 (n = 5; *in vitro* ICI) received 2 doses of the Pfizer-BioNTech COVID-19 vaccine (mRNA vaccine). Separately, three patients who had received COVID-19 vaccinations or experienced natural SARS-CoV-2 infection prior to ICI treatment were recruited from the Clatterbridge Cancer Center in Liverpool, UK (HYST, ethics approval ref 12/NW/0525). Blood samples were obtained, and PBMCs were isolated from the three patients prior to, 4 weeks and 8 weeks following initiation of ICI treatment. Only samples from patients who did not receive a COVID-19 vaccination or present with or report natural SARS-CoV-2 infection during the sample collection timeline were utilized in the assays. Blood samples from healthy volunteers (vaccinated/vaccine naïve) were collected through the Liverpool Pharmacology Biobank (UoL11499). All volunteers in this study gave written and informed consent and ethical approval for all studies was obtained from the Liverpool Research Ethics Committee in accordance with the Declaration of Helsinki.

### Lymphocyte transformation test

LTTs were conducted as outlined by Pichler and Tilch^9^ with PBMC (1.5 × 10^5^ cells/well) from ICI-treated patients (*in vivo* ICI) and healthy donors (*in vitro* ICI), incubated with antigens for 7 d. Healthy donor PBMC was treated with and without checkpoint blockade antibodies *in vitro*; anti-PD-1 mAb (clone; A17188B) or anti-PD-L1 mAb (clone; 29E.2A3) (5 µg/mL; BioLegend, UK). Healthy donor PBMC (n = 4) were incubated with CEF(T) peptide pools (0.125–0.5 ug/mL; ProImmune Ltd, Oxford, UK) ± PD-1 and PD-L1 block for 7 d. Healthy donor PBMC was isolated from donors 14 d post their first COVID-19 vaccination (n = 5), 28 d post their second COVID-19 vaccination (n = 3) and 6 months post their second COVID-19 vaccination (n = 5) and incubated with wild-type SARS-CoV-2 spike pool 1 and spike pool 2 (0.25–1 µg/mL) ± PD-1 and PD-L1 block for 7 d. T-cell responses to SARS-CoV-2 spike proteins were assessed using overlapping peptide pools comprised of 15-mer peptides with an 11-amino-acid overlap spanning the N-terminal of the S1 and S2 domains. PBMC samples taken from clinical visits of oncology patients (atezolizumab (n = 1); pembrolizumab (n = 1); nivolumab (n = 1)) prior to and 4 weeks (n = 3) and 8 weeks (n = 1) were obtained following initiation of ICI-treatment to investigate recall responses to an antigen panel. Patient PBMC were incubated with wild-type SARS-CoV-2 spike pool 1 and spike pool 2 (0.25–1 µM) or ChAdOx1 nCoV-19 (1,000 viral particles (VP)/cell; AstraZeneca, UK) for 7 d. On day 6 of the LTT assay, [^3^H]-thymidine was added (0.5 μCi/well) and cultures were incubated for an additional 16 hours. Proliferation was assessed via [^3^H]-thymidine incorporation and given as counts per minute (CPM) + standard deviation. Proliferation data is presented as Stimulation Index (SI) values (average cpm values of treated cultures/average cpm values of control cultures).

### Evaluation of cytokine and cytolytic molecule secretion in PBMC cultures

Cytokines and cytolytic molecules were quantified from PBMC culture supernatant (25 μL) using bead-based immunoassays according to the manufacturer’s instructions (LEGENDplex, BioLegend Custom Human 11-plex panel (granzyme B, IFN-γ, IL-10, IL-13, IL-22, IL-5, IL-6, perforin, sFasL, IL-17A, TNF-α). Secretion of cytokines below the threshold of 5 pg/mL was not included in the analysis.

### Statistical analysis

Statistical analysis was performed using a two-way ANOVA test using GraphPad Prism 10.5. For these tests, *p < .05 was deemed the threshold for statistical significance (*p < .05, **p < .01, ***p < .001, ****p < .0001).

## Results

### Memory T-cell responses to CEF(T) were enhanced with in vitro immune checkpoint blockade in healthy donors

To investigate whether immune checkpoint blockade enhanced memory T-cell responses to antigens, LTTs were carried out in healthy donors with the model antigen CEF and CEFT peptide pools together in one pool (CEF(T)) under standard (no ICI block) conditions or with *in vitro* PD-1 or PD-L1 inhibition. Dose-dependent proliferative responses were detected to the CEF(T) pool in 4/4 donors. With *in vitro* PD-1 blockade, the proliferative response to CEF (T) pools was increased compared to standard conditions in 3/4 donors, reaching statistical significance for donor 1 (***p < .001) and donor 2 (**p < .005) (Figure 1A). With *in vitro* PD-L1 blockade, the proliferative response to CEF (T) pools was also increased compared to standard conditions in 3/4 donors, with statistical significance reached for donor 2 (*p < .05) (Figure 1A). This observed variability may reflect differential expression of PD-1/PD-L1 alongside the functional dependence of each healthy volunteer on this co-inhibitory pathway. To this end, the role of PD-1/L-1 expression within response variability was further assessed in the context of recall responses to CEF(T) using PBMCs collected from healthy volunteers. PD-1 and PD-L1 expression was measured at baseline (day 0) and following peptide simulation (day 7), with corresponding proliferation-based data collected to enable associations to be made between donor PD-1/L-1 expression and response magnitude (stimulation index) after CEF(T) exposure (Supplementary Figure S1). PBMC from healthy donors (n = 3) were assessed for T-cell proliferation within LTT assays, and varying orders of recall response intensity were observed (Donor 1, SI = 2.4; Donor 2, SI = 15; Donor 3, SI = 27). Interestingly, basal PD-1 and PD-L1 expression was observed to be reduced in PBMC cultures pertaining to donors with a greater magnitude of recall response to CEF(T). Conversely, PD-1 and PD-L1 expression was found to be increased on day 7 in donors capable of producing high magnitude CEF(T)-specific T-cell responses. Furthermore, the strength of the initial recall response and preexisting CEF(T)-specific immunological memory may influence the magnitude of response enhancement achieved with immune checkpoint blockade.

**Figure 1.**
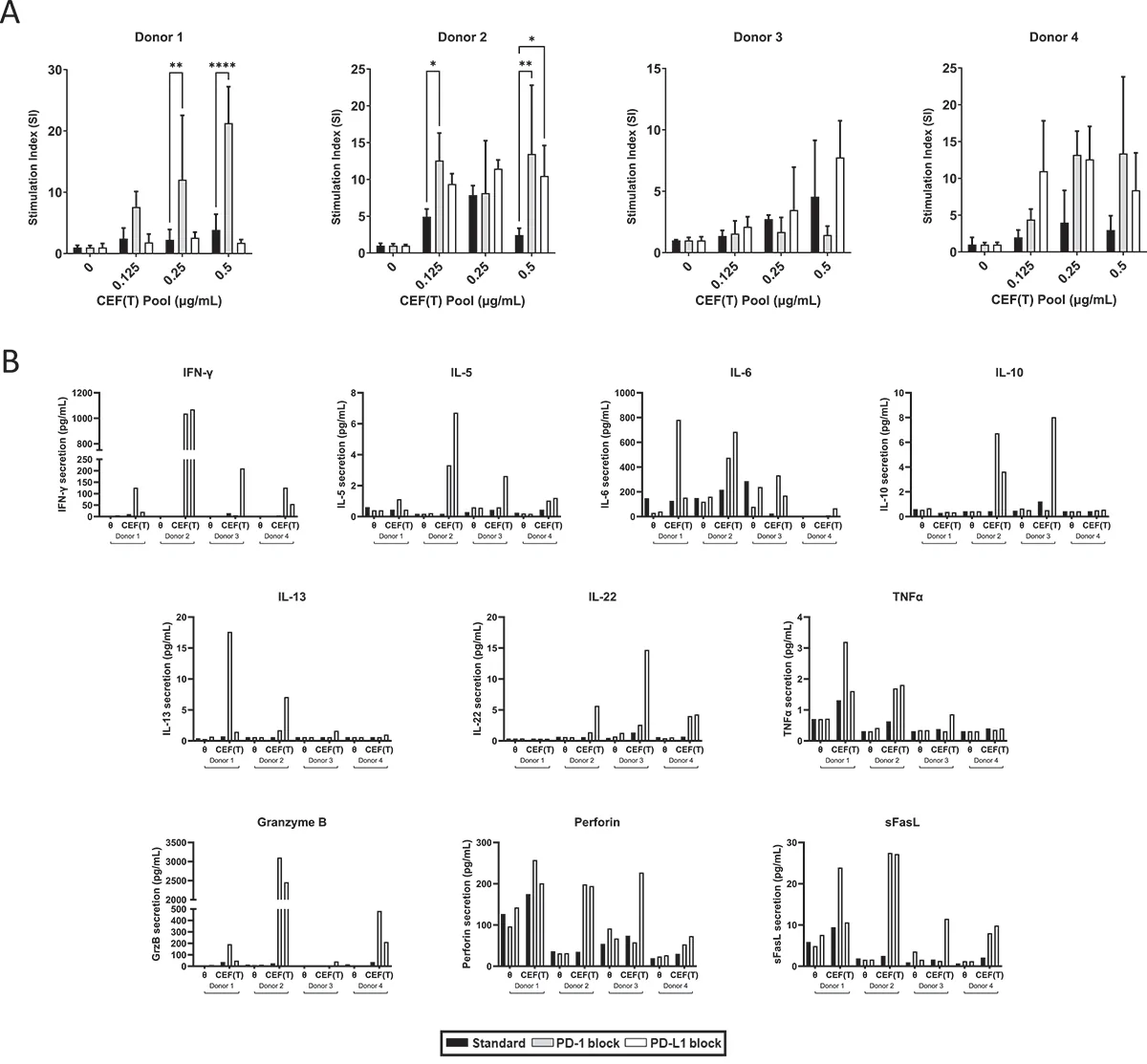
*In vitro* PD-1 and PD-L1 blockade enhanced PBMC proliferative responses and cytokine and cytolytic molecule secretion in response to CEF(T) peptide pools in four healthy donors. (A) PBMC (1.5 × 10^5^ cells/well) were cultured with R9 medium (standard conditions) or PD-1 or PD-L1 monoclonal antibodies (5 µg/mL) and either R9 medium (negative control) or CEF(T) peptide pool (0.125–0.5 µg/mL) and incubated for 7 d. Proliferation was measured via [^3^H]-thymidine incorporation (CPM values) and is presented as SI values + standard deviation. Statistical significance compared to media-treated control was determined using Dunnett’s multiple comparisons two-way ANOVA test (*p < .05, **p < .01, ***p < .001, ****p < .0001). (B) Supernatants from triplicate test cultures of the R9 medium (negative control) and CEF(T) peptide pool (0.5 µg/mL) wells were pooled in equal volumes (25 µL) and utilized in the LEGENDplex assay to determine the mean secretion of IFN-γ, IL-5, IL-6, IL-10, IL-13, IL-22, TNF-⍺, granzyme B, perforin, and sFasl in singlet on each pooled sample.

Supernatant pools (25 µL/well) from the negative control and CEF (T) pool (0.5 µg/mL) were collected from test cultures immediately prior to the addition of tritiated thymidine and subject to quantitative analysis of cytokine and cytolytic molecule secretion. Cytokine bead array profiling revealed that under standard conditions, the secretion of effector molecules (>1.5x basal levels) was limited to perforin (2/4 individuals), and sFasL and granzyme B were detected in (1/4 individuals) (Figure 1B). Following *in vitro* PD-1 or PD-L1 inhibition, a broader and intensified mediator release was observed in the same individuals (Figure 1B).

### Memory T-cell responses to SARS-CoV-2 spike 1 and 2 peptide pools were enhanced with in vitro immune checkpoint blockade in healthy donors

To evaluate memory T-cell responses to SARS-CoV-2 pools 1 and 2, LTTs were conducted on PBMCs sampled from healthy donors longitudinally at timepoints after COVID-19 vaccination (14 d post COVID-19 first vaccination, 28 d post COVID-19 second vaccination and 6 months post COVID-19 second vaccination). In addition to the conduction of these LTTs, parallel cultures were set up in which *in vitro* immune checkpoint inhibition (PD-1/PD-L1 blockade) was applied. Proliferative responses above baseline were detected to SARS-CoV-2 wild-type spike pools 1 and 2 (0.25–1 µg/mL) in healthy donors at all time points following vaccination (Figure 2A–C). Significant enhancement in PBMC proliferative responses to SARS-CoV-2 wild-type spike pools 1 or 2 were observed with *in vitro* immune checkpoint blockade in most individuals at all visitations. Furthermore, baseline proliferation of PBMC cultures collected from vaccine naïve healthy donors were unaffected after exposure to spike pools 1/2 in the presence and absence of PD-1 blockade (Figure 2D). Additional experiments were conducted to demonstrate that recall responses to the model antigens, CEF(T) and SARS-CoV-2 spike pools, were consistent between LTT assays (7 d) and cytokine release assays (IFNγ;48 h) (Supplementary Figure S2).

**Figure 2.**
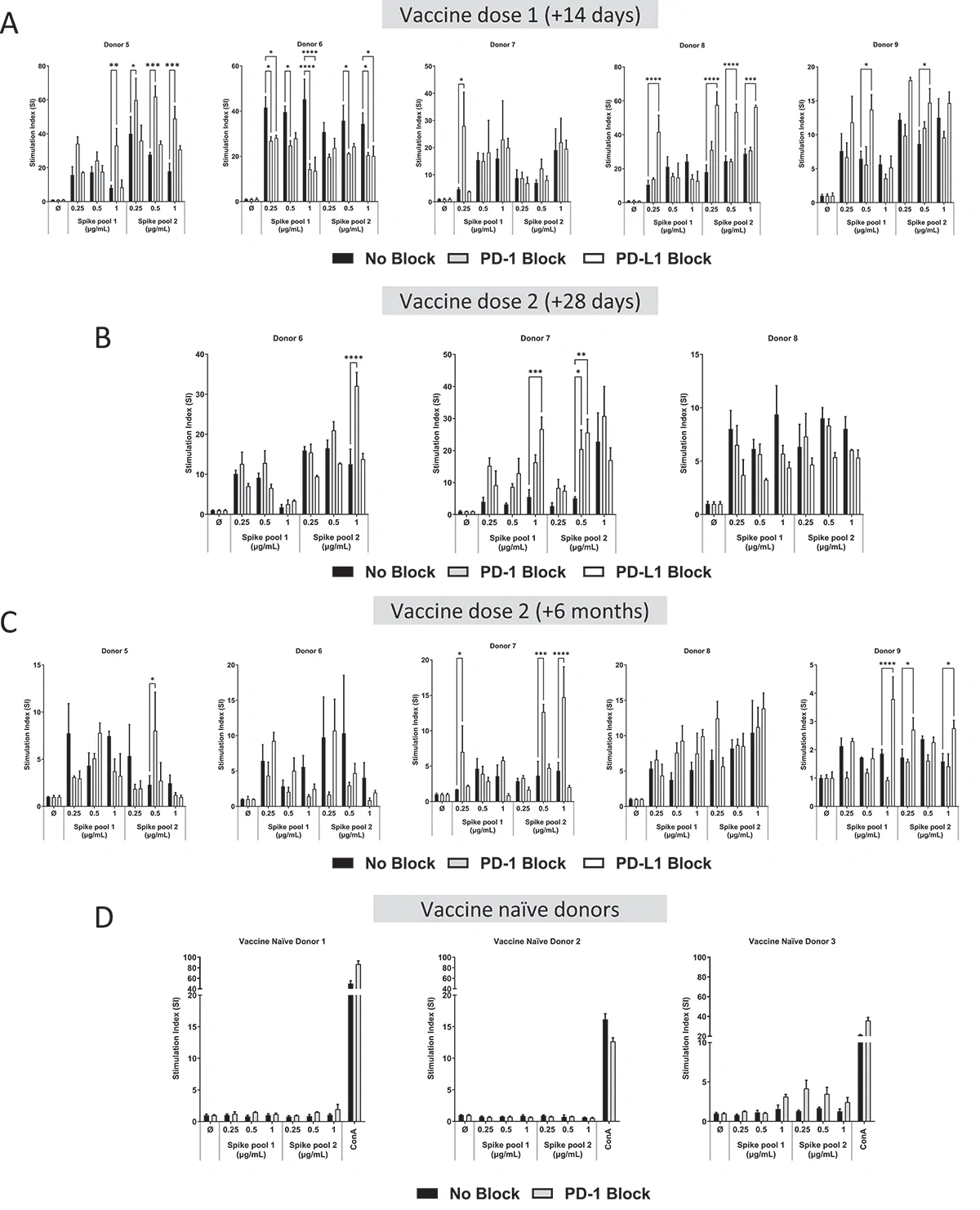
*In vitro* PD-1 and PD-L1 blockade enhanced PBMC proliferative responses to wild-type SARS-CoV-2 spike peptide pools 1 and 2 in healthy donors at various timepoints after COVID-19 vaccination. PBMC (1.5 × 10^5^ cells/well) were cultured with R9 medium (standard conditions) or PD-1 or PD-L1 monoclonal antibodies (5 µg/mL) and either R9 medium (negative control) or SARS-CoV-2 spike pool 1 (0.25–1 µg/mL) or SARS-COV-2 spike pool 2 (0.25–1 µg/mL) and incubated for 7 d. Proliferation was measured via [^3^H]-thymidine incorporation (CPM values) and is presented as SI values + standard deviation. (A) PBMC from five healthy donors, 14 d post-vaccine 1. (B) PBMC from three healthy donors, 28 d post-vaccine 2. (C) PBMC from five healthy donors, 6 months post vaccine 2. (D) PBMC from three vaccine naïve healthy donors. Statistical significance compared to media-treated control was determined using Dunnett’s multiple comparisons two-way ANOVA test (*p < .05, **p < .01, ***p < .001, ****p < .0001).

### Memory T-cell responses to COVID spike pools were enhanced with in vivo immune checkpoint blockade in IO patients

Three immune-oncology (IO) patients (Patient 1, Non-small cell lung cancer; Patient 2, Head and neck cancer; Patient 3, Renal cancer) with a history of COVID-19 vaccination or SARS-CoV-2 natural infection were recruited and subjected to serial blood sampling before and after their IO treatment (Figure 3A-C). Demographic and clinical information relating to the patients studied can be viewed in Supplementary Table 1. To investigate how *in vivo* immune checkpoint blockade would impact memory responses to antigens, IO patients were recruited after natural SARS-CoV-2 infection or after COVID-19 vaccination. Thus, all patients were expected to possess established memory T-cell responses to SARS-CoV-2 spike 1 and 2 peptides prior to ICI administration. Clinical records for IO patients were maintained, with any incidence of SARS-CoV-2 natural infections or COVID-19 vaccinations between the timepoints of their pre- and post-IO PBMC sample acquisition recorded. Due to patients 2 and 3 receiving COVID-19 vaccinations after their visit 2 sample acquisition and before visit 3 sample acquisition, only pre-IO (visit 1) and one post IO (visit 2) sample qualified for use in this study (Figure 3B-C).

**Figure 3.**
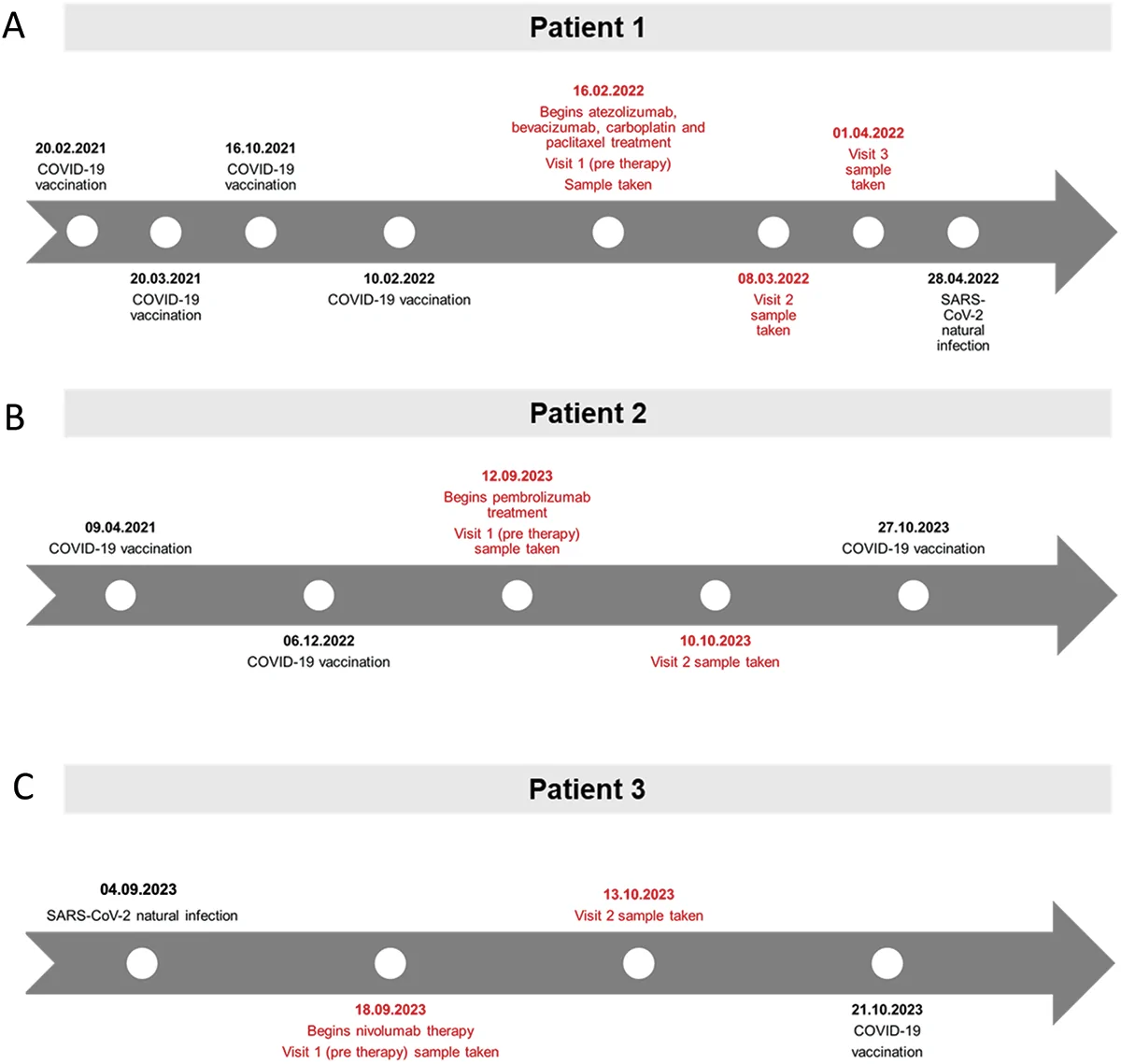
Timeline of SARS-CoV-2 natural infections, COVID-19 vaccinations, administration of IO treatment and sample acquisition dates from three ICI-treated patients. (A) Patient 1 (B) Patient 2 (C) Patient 3.

LTTs were conducted on patient PBMCs before and after clinical administration of immune checkpoint blockade (Patient 1, atezolizumab; Patient 2, pembrolizumab; Patient 3, nivolumab). Responses were evaluated to SARS-CoV-2 spike peptide pools 1 and 2 (0.25–1 µg/mL), ChAdOx1 nCoV-19 (1,000 VP/cell) and concanavalin A (ConA; 5 µg/mL) as the positive control. Enhanced PBMC proliferative responses were observed in response to wild-type SARS-CoV-2 spike pools 1 or 2 post-ICI treatment for patients 1, 2 and 3, and proliferative *in vitro* responses to the ChAdOx1 nCoV-19 vaccine were increased post-ICI treatment for patient 1 (Figure 4A). Importantly, as depicted in Figure 4B, the absolute proliferative capacity of cultures pre- and post-immunotherapy was demonstrated to be comparable through utilization of nonspecific mitogen stimulation of cultures.

**Figure 4.**
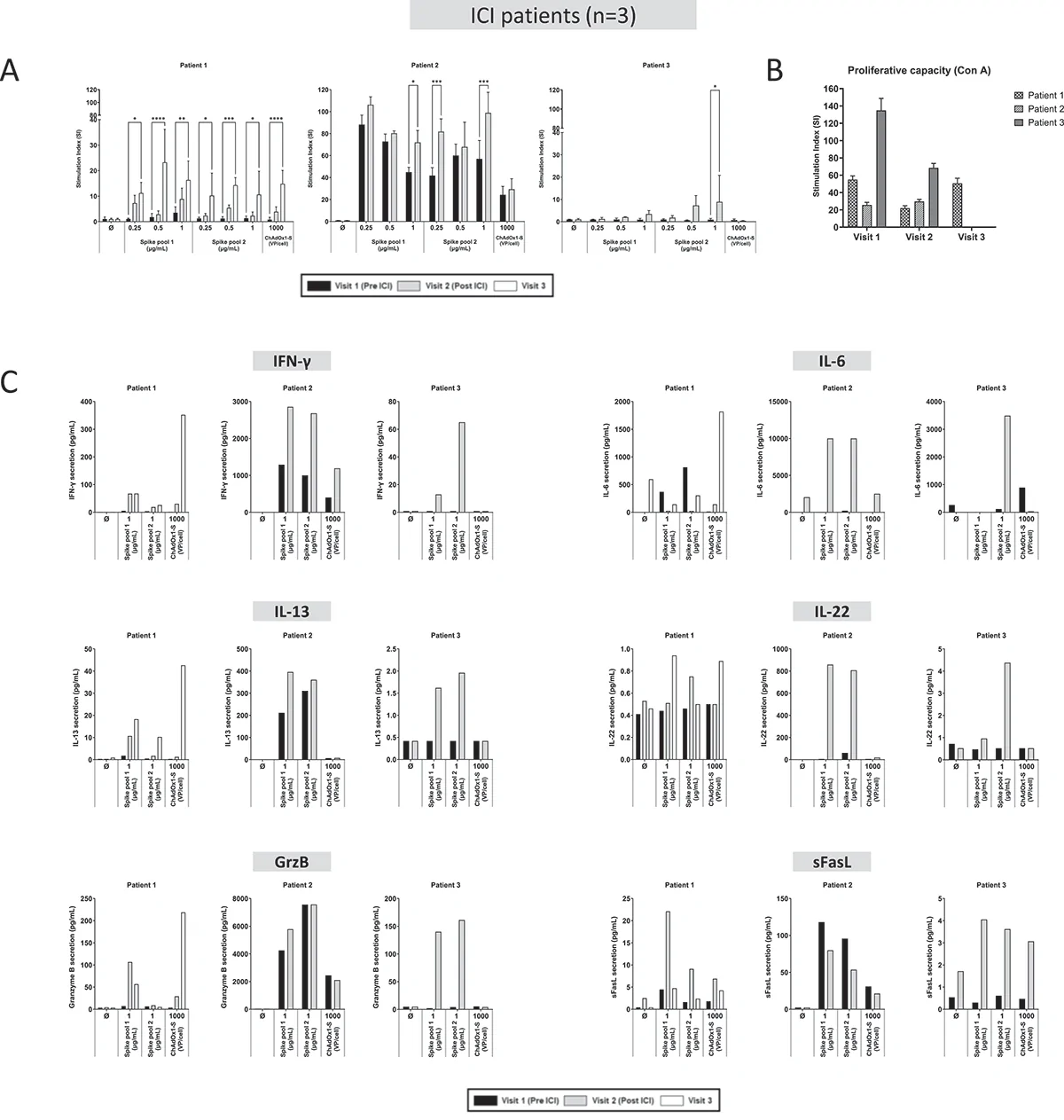
PBMC from three patients post-ICI treatment proliferated and secreted cytokines and cytolytic molecules in response to wild-type SARS-CoV-2 spike peptide pools 1 and 2 and ChAdOx1 nCoV-19 vaccine. (A) PBMC (1.5 × 10^5^ cells/well) were cultured for 7 d with either R9 medium (negative control) with (A) wild-type SARS-CoV-2 spike pool 1 (0.25–1 µg/mL), wild-type SARS-CoV-2 spike pool 2 (0.25–1 µg/mL), ChAdOx1 nCoV-19 vaccine (1,000 viral particles (VP)/ cell) or (B) Concanavalin a (con A; 5 µg/mL) (C) Supernatants from triplicate test cultures of the R9 medium (negative control), wild-type SARS-CoV-2 spike pool 1 (1 µg/mL), wild-type SARS-CoV-2 spike pool 2 (1 µg/mL) and ChAdOx1 nCoV-19 vaccine (1,000 viral particles (VP)/ cell) wells were pooled in equal volumes (25 µL) and utilized in the LEGENDplex assay to determine the mean secretion of IFN-γ, IL-6, IL-13, IL-22, granzyme B, and sFasl in singlet on each pooled sample.

Supernatant pools (25 µL/well) from the negative control, wild-type SARS-CoV-2 spike peptide pools 1 and 2 (1 µg/mL) and ChAdOx1 nCoV-19 (1,000 VP/cell) conditions were collected from test cultures immediately prior to the addition of tritiated thymidine and used to quantify cytokine and cytolytic molecule secretion for visit 1 (pre-immunotherapy) and post-immunotherapy (visit 2 and 3 for patient 1, visit 2 for patients 2 and 3). Cytokine bead array profiling revealed that the secretion of cytokines and cytolytic molecules ≥1.5-fold relative to the negative control occurred at varying rates in response to SARS-CoV-2‑related antigens and ChAdOx1 nCoV-19 between all patients across visits, with a general observed enhancement of cytokine responses post-immune checkpoint blockade therapy (Figure 4B). Increases in IFN-γ secretion were detected post-*in vivo* immune checkpoint blockade across all three patients in response to SARS-CoV-2 spike pool 1 and 2, and in response to ChAdOx1-S vaccine for patients 1 and 2 (Figure 4B). Secretion of IL-6, IL-22 and granzyme B was sporadically (though robustly when it occurred) increased for antigens across patients.

## Discussion

This study details the evaluation of recall lymphocyte responses to classic and SARS-CoV-2 relevant antigens following *in vitro* and *in vivo* PD-1 pathway checkpoint inhibitor treatment. We observed enhancement of responses in terms of proliferative responses and cytokine/cytolytic molecule secretion following *in vitro* PD-1/PD-L1 blockade in PBMC derived from healthy volunteers. The recall responses to spike pools 1/2 are likely a direct result of transgene (spike) expression and immunological memory following vaccination and/or natural infection, as such responses were absent in vaccine naïve groups. This augmentation of memory responses with the checkpoint inhibitors to the panel of antigens was also seen in the responses from cancer patients following clinical administration of PD-1/PD-L1 blockade agents. These findings are in line with the expected pharmacology of this class of pharmacological agent, and this expected enhanced immunological response has been a subject of consideration with respect to efficacy and safety of vaccination in patients on an established ICI treatment regimen.

To contextualize our findings, a short literature review was conducted to find relevant studies that detailed the safety/efficacy profile of COVID-19 vaccination in ICI treated patients and specifically focused on studies that evaluated T-cell responses within these individuals (Table 1). To summarize, studies have now extensively evaluated the bidirectional impact of concomitant prophylactic vaccinations for influenza and COVID-19 alongside checkpoint inhibitor treatment on safety and efficacy of each therapeutic.^27,28^ Historical concerns over generalized higher rates of immune-related adverse events (irAEs) in ICI treated patients,^7^ have largely been allayed with more substantive studies and pooled analysis. In terms of evaluating T-cell responses, the bulk of the available literature focused on patients established on an ICI therapy regimen prior to vaccine administration.^18,22^ In general, most of the studies reviewed (Table 1) focus on the efficacy and safety in patients receiving established IO treatment regimens who were vaccinated, whereas for the three index patients in this report, the explicit focus has been how ICI treatment may influence a rechallenge event. Indeed, in this study natural SARS-CoV-2 infection and COVID-19 vaccination were recorded for ICI-treated patients; therefore, only patients with no recorded natural SARS-CoV-2 infection or COVID-19 vaccinations once ICI treatment had commenced were utilized in these assays. This was to assure as much as possible that rechallenge events were not influenced by relevant immunological challenges to the studied antigens in the interim period; the possibility of unreported natural COVID-19 infection is therefore considered low but cannot be excluded. A similar approach was undertaken by Piening et al.^24^ and found no enhancement of humoral or T-cell responses when analyzing across a population, which might be interpreted as in contrast to our own findings. However, while we report enhancement for multiple antigens with both *in vitro* and *in vivo* ICI administration, overt inter-individual variability was observed. An in-depth evaluation of the Piening et al.^24^ study reveals similar inter-individual variability, with enhancement of cellular responses commonly observed. With respect to individuals who exhibited apparent reductions in cellular responses in that study, the authors acknowledge a lack of data on chemotherapeutic/immunosuppressive drug use, and shortcomings in infection/vaccination clinical record keeping may further convolute interpretation of data. Furthermore, the interval between sampling is more protracted in the Piening study relative to ours. We therefore conclude that the two studies are complementary, and that the general impact of ICI administration on recall responses to the evaluated antigens is augmentation, but that inter-individual variability, along with dynamic patient‑specific factors, can preclude its clear detection across a population.

**Table 1.** Clinical and mechanistic studies detailing the impact of ICI therapy on the safety and efficacy of COVID-19 vaccination.

Study	Key clinical outcomes	Functional evaluation	Reference
**Clinical studies**			
Meta-analysis evaluating some 27 individual studies, a total of 8331 patients (of which 4724 received ICIs). Vaccines used are mostly mRNA vaccines (BNT162b2 or mRNA-1273).	No significant elevation of irAE incidence or severity after vaccination.	No significant enhancement of seroconversion in ICI treated individual’s vs. controls without cancer, though enhancements seen relative to those on chemotherapy. Slight enhancement of T-cell response rate.	^10^
394 lung cancer patients receiving (n = 150) or not receiving (n = 244) COVID-19 vaccination (not specified but mix of inactivated and mRNA vaccines).	No clear impact on PFS or irAE incidence/severity.	None reported.	^11^
64 patients with various tumors vaccinated with BNT162b2, mRNA-1273 and AZD1222.	No significant difference in irAEs in vaccinated group relative to non-vaccinated or historical groups. Enhancement of survival in vaccinated group. No clinically diagnosed CRS.	Elevated cytokines observed in a subset of patients. However, cytokine samples taken up to 6 weeks after vaccination.	^12^
Retrospective analysis of 81 cancer patients receiving ICI therapy. Cancer subtypes included lung (27%), melanoma (23%), kidney cancer (14%), and gastrointestinal/hepatic cancer (11%). Most patients received pembrolizumab (56%) or nivolumab (27%). Patients received either the BNT162b2 (83%) or mRNA-1273 (17%) COVID-19 vaccines.	After a minimum of 30 d of follow-up from the second vaccine dose, 0/84 patients had an exacerbation of a previous irAEs or diagnosis of new irAEs.	Observational study only, no functional evaluation or mechanistic profiling. Retrospective chart review was performed using electronic medical records. Study cohort included cancer patients who received ICIs within one month of receiving a COVID-19 mRNA vaccine.	^13^
Retrospective study of 284 patients with solid tumors receiving ICI therapy (nivolumab and ipilimumab). Incidence of severe (irAEs) in cancer subjects treated with ICIs studies following COVID-19 vaccination. Individual vaccine platforms were not included in analysis.	29 patients (10.2%) developed severe irAEs. Of these, 12 patients (41.4%) received ICI monotherapy, and 10 patients (34.5%) received combination ICI therapy with nivolumab and ipilimumab. In patients who experienced severe irAEs, only 5 (17.2%) experienced a severe irAE after the first vaccine dose. The majority of severe irAEs occurred after the second (15 patients, 51.7%) and third (9 patients, 30%) vaccine doses.	Observational study only, no functional evaluation or mechanistic profiling. Data was obtained from review of electronic medical records and included baseline demographics, cancer treatment history, COVID-19 vaccination history, and the occurrence of severe immune-related events.	^14^
32 patients with mixed tumor types, majority receiving nivolumab, and multiple patients receiving atezolizumab, compared to unvaccinated subgroup and healthy controls. Vaccines administered were Sinovac and Pfizer-BioNTech up to 5 doses.	No clear evidence of vaccine related enhancement of irAEs or efficacy with either vaccine. Reactive lymph node enlargement following vaccination associated with positive prognostic outcome.	No significant difference in antibody response to vaccine in ICI treated groups relative to control group, though possible evidence of ICI modulated baseline enhancement.	^15^
408 patients with cancer received ICI therapy, mainly pembrolizumab (65%) and nivolumab (24%). Incidence of irAEs was assessed after the administration of a COVID-19 mRNA vaccine (BNT162b2) within 90 d of ICI treatment.	ICI treatment was not associated with an increased incidence of irAEs. This extended to individuals with a clinical history of irAEs arising from ICI therapy. No increased risk of COVID-19 diagnosis or breakthrough infection was observed following ICI therapy and vaccination.	Observational study only, no functional evaluation or mechanistic profiling.	^16^
**Functional and mechanistic studies**			
Comparison of COVID mRNA vaccination vs. no vaccination [(NSCLC (n = 704 vs. n = 132) and melanoma (n = 167 vs. n = 43)]. BNT162b2 or mRNA-1273 administered.	Greater OS in mRNA vaccinated patients. *Though contested based on time zero bias.^17^	Murine *In vivo* models corroborate greater antitumour activity. Blockade and recombinant cytokine experiments demonstrated this is due to type I IFN elicited by vaccines. Cytokine elevations demonstrated in healthy volunteers administered vaccine.	^6^
Longitudinal study on 88 patients with cancer treated with (PD-(L)1 inhibitors. BNT162b2 (two doses) No comparator control group in initial study. Small chemoimmunotherapy combination group included for extension.	No infections or significant safety findings.	ICI treated patients experienced and naïve to SARS-CoV-2 exhibited seroconversion (S1/S2 titers and neutralizing antibodies) and increase in spike peptide pool responsive T-cells. 6 month follow up in 60 patients showed slight waning of responses. Chemoimmunotherapy associated with inferior responses.	^18^ ^19^
Prospective, multi-center study evaluating multiple groups; A) Non-cancer (n = 246) B) PD-1/PD-L1 immunotherapy (n = 136) C) cytotoxic chemotherapy (n = 236) D) chemoimmunotherapy (n = 147). Patients received 2 doses of mRNA-1273.	Non-inferiority of immunotherapy group relative to control with respect to vaccine efficacy. No clear imbalance in the incidence or severity of irAEs. One incidence of erythema multiforme (1^st^ dose of vaccine) which developed to SJS following 2^nd^ dose.	Humoral (S1 IgG) assessment at pre- 1^st^ and 2^nd^ vaccination timepoints, and 28 d post second vaccine. Cellular response (S1 and S2 peptide pools) at baseline, and 28 d and 6 months post second vaccine. Similar S1 IgG titers and spike specific T-cell responses achieved in immunotherapy group relative to non-cancer group.	^20^
Phase IV prospective multicentric trial in patients (204) with solid cancer and a reference group of 73 healthy controls. Patients treated with a variety of distinct agents (TKI, mAb, ICI, CT and CT/mAb combination). BNT162b2 vaccine (30 µg 2 doses).	No clear evidence to contraindicate vaccination in ICI group.	Humoral (anti-S antibody) and cellular (T-cell responses to S1/S2 antigens) monitored longitudinally. Comparable IFNγ T-cell response (to S1 and S2 CD4 and CD8 epitopes) and anti-S antibody titer generation following vaccination over the time course of 3 months in ICI treated group to most other cancer patient cohorts.	^21^
Prospective observational study; 20 NSCLC patients receiving ICIs (PD-1/PD-L1 blocking agents with or without chemotherapy) vs. 38 controls (healthcare workers). Vaccines used were BNT162b2 or ChAdOx1-S (patients) or BNT162b2 for up to 3 doses.	Limited reporting.	Humoral (serum IgG to the RBD) and cellular (T-cell responses to S1 spike peptides) monitored longitudinally. Modest decrease in IgG and T-cell responses initially (after 2 but not 3 doses) in patient subgroup. Effect may be driven by chemotherapeutic agents.	^22^
Evaluation of 22 healthy donors and 118 patients with cancer including subgroups treated with immunotherapy (n = 36, of which 34 were on PD-L1/PD-1 or CTLA-4 ICIs), chemotherapy (n = 15) and chemoimmunotherapy (n = 6). Vaccination with mRNA-1273 or BNT162b2 for at least 2 doses.	Adrenal crisis occurred in two patients with history of adrenal insufficiency.	Humoral (serum IgG to spike and the RBD) and cellular (T-cell responses to Spike protein peptide pools derived from WT or B.1.617.2 variant) monitored longitudinally. Similar, though more variable antibody titers to spike and RBD between anti-PD-1 immunotherapy group and healthy donors. T-cell responses to spike pool comparable between IO and HD groups and variant cross-reactivity was observed in around 50% of individuals.	^23^
Cohort of 29 patients previously vaccinated initiating therapy with ICI (PD-1/PD-L1 blocking agents; pembrolizumab, durvalumab, nivolumab and atezolizumab), compared to 12 unvaccinated individuals over a similar time period.	Limited reporting.	Humoral (serum IgG to the spike protein) and cellular (T-cell responses (spike protein derived peptide pool) monitored longitudinally – prior to and 2–3 weeks after the second infusion of ICI). No reliable enhancement in antibody titers observed in the ICI treated group relative to baseline. Limited enhancement of spike specific T-cell responses relative to baseline (not significant across a population, but instances of apparently enhanced responses).	^24^
Cohort of 200 cancer patients (ICI therapy; n = 31) assessed for seroconversion rates (anti-spike IgG titer) post vaccination) following COVID-19 vaccination with both adenoviral and mRNA-based platforms.	30/31 (97%) of cancer patients receiving ICI therapy demonstrated high seroconversion post vaccination. Lower IgG titers were observed after vaccination with adenoviral-based vaccines (AD26.COV2.S) compared with mRNA COVID-19 vaccine (BNT162b2 and mRNA-1273).	Anti-spike protein IgG antibody titers following COVID-19 vaccination did not significantly differ in patients receiving ICI therapy when compared with vaccinated cancer patients who were not receiving ICI therapy. Study did not evaluate the level and magnitude of SARS-CoV-2-specific T-cell responses in ICI patients following vaccination.	^25^
Cohort of 524 patients with solid and hematologic cancer. 24 patients received immunotherapy (anti-PDL1/PD1 or anti-CTLA-4). Seropositivity rate for anti-S IgG 36 d after vaccination with either mRNA-1273 or BNT162b2. SARS-CoV-2-specific CD8+ and CD4+ T cell responses following mRNA vaccination in patients with solid and hematologic malignancies were also evaluated in non-ICI patients.	No evidence of vaccine related enhancement of irAEs or efficacy with either vaccine. Across the wider cohort (n = 524), patients with solid cancer elicited strong humoral immune responses following the administration of COVID-19 mRNA vaccines.	In patients receiving standard immunotherapy with anti-PDL1/PD1 or anti-CTLA-4, only 1 of 24 was seronegative for anti-S IgG 36 d after vaccination with mRNA-based COVID-19 vaccines.	^26^

## Conclusion

Within the small cohort we evaluated, vaccination was not associated with adverse events in patients, and the study would in any case be insufficiently powered to draw conclusions about whether this *ex vivo* assay is informative with regard to potential adverse reactions after SARS-CoV-2 infection or COVID-19 vaccination.^29^ Similarly, while overt increases in T-cell responses are observed to pathogen‑specific antigens derived from classical sources, the clinical correlate of afforded protection cannot be determined. Due to the size of the study cohort, HLA diversity represents an additional limiting factor as the allelic representation of patients and healthy donors does not capture the full range of HLA-restricted peptide-binding present across broader populations. Furthermore, the deployment of CEF(T) as a model recall antigen across the donor panel cannot account for the heterogenous nature of preexisting immunity to viral peptides and likely contributes to the inter-individual variability observed within our assays. However, it is of interest to note that there is variability in the response to ICI modulation of patient and healthy donor T-cell responses in terms of the intensity and qualitative nature observed, and that this variability likely reflects the heterogeneity of patient T-cell compartments. The breadth of cytokine analysis presented here offers a representation of the qualitative diversity of T-cell responses, several of which have demonstrable ICI dependency. This diversity may offer an explanation for differentiated clinical responses at the individual level. Further studies are required to understand the role of such responses in patients presenting with rare deleterious reactions following concomitant vaccination/ICI treatment, for which T-cell characterization at both population and clonal levels may prove fruitful.

## Supplementary Material

260044104 R1 Supplementary Table.docx

Supplementary Figure 1.JPG

260044104 R1 Supplementary Methods.docx

Supplementary Figure 2.JPG

## Data Availability

Datasets related to this article are available upon request from the corresponding author.
