# Supplementary material for: Immune checkpoint inhibition enhances memory T-cell responses to CEF(T), SARS-CoV-2 spike proteins and ChAdOx1 nCoV-19
Source: Hum Vaccin Immunother. 2026 Oct 6;22(1):2742035. doi: 10.1080/21645515.2026.2742035 (PMC13644633; doi:10.1080/21645515.2026.2742035)
Supplement: Supplementary file 1 [file KHVI_A_2742035_SM9820.docx]

**SUPPLEMENTARY METHODS**

**Isolation of peripheral blood mononuclear cells**

Whole venous blood was collected using vacutainer heparinised tubes from drug naïve healthy volunteers and PBMCs isolated by density gradient centrifugation. Briefly, whole blood was layered over an equal volume of Lymphoprep™ (STEMCELL™ Technologies) and centrifuged at 2000 rpm for 25 minutes. The resultant buffy coat, containing the lymphocyte fraction, was collected and washed twice with Hanks’ Balanced Salt solution (HBSS) before resuspension in RPMI-based T-cell culture medium supplemented with penicillin (100 μg/mL), streptomycin (100 U/mL), holo-transferrin (12.5 mg/mL), HEPES (25 mM), L-glutamine (2 mM), and 5% human AB serum. Cell counts and viability were determined using a Neubauer haemocytometer after staining with trypan blue (0.2 % w/v).

**Enzyme-linked immunospot assay**

Cytokine release (IFNγ) from CEF(T) or spike protein peptide pool exposed PBMC cultures was detected by enzyme-linked immunospot (ELISpot) assays. Activated (35% ethanol) Protein Binding Immobilon-P Membrane 96-well Multiscreen® filter plates were coated with 100 μL of IFNγ (Cat. No 3420-3, 1:100 dilution) diluted in sterile HBSS according to the manufacturer’s instructions (Mabtech, Nacka Strand, Sweden). Coated plates were then incubated for 24 h at 4 °C. PBMCs from healthy donors, vaccine naïve donors and vaccinated donors were washed in RPMI-based T-cell culture medium to exclude any cytokine milieu and seeded at 2x10^5^ cells/well. PBMCs were incubated in pre-coated Multiscreen® filter plates with CEF(T) peptide pools (1 µg/mL) or spike (S) pools (1 µg/mL) for 48 h. For assay development, cells were discarded, and ELISpot plates were washed (x5) with HBSS. Detection antibodies were conjugated with biotin corresponding to the cytokines of interest and diluted in HBSS supplemented with 0.5% FBS. Biotinylated antibody solution was added to each well and incubated for 2 h. Following incubation, plates were washed with HBSS and streptavidin conjugated alkaline phosphatase (1:1000 dilution) was added and plates were incubated for 1 h. BCIP-NBT substrate solution was added to the assay and plates were incubated at RT for 15–20 min in the dark to allow colorimetric reaction to occur. Spots were imaged using an AID ELISpot reader (Cadima Madical, Stourbridge, UK).

**Flow cytometry and surface marker expression**

Surface expression of co-inhibitory (PD-1 and PD-L1) molecules on PBMC populations were acquired by flow cytometry. To identify co-inhibitory PD-1 and PD-L1 expression for individual immune-cell populations, PBMCs collected from healthy drug naïve donors (n=3) were exposed to CEF(T) peptide pools (1 µg/mL) or media and stained in triplicate with fluorophore-conjugated antibodies, including Live-dead (Fixable Near-IR, Cat. No. L34975), PD-1 (BD Biosciences; Cat. No. 567617) and PD-L1 (BD Biosciences; Cat. No. 568319) at day 0 and 7. Antibodies were used at concentrations recommended by the manufacturer, and staining procedures were performed in accordance with the manufacturer’s instructions. Fluorescence was measured from the recorded 100,000 events per sample using the BD FACSCanto II and FlowJo® version 10 software was used for data analysis. Cell expression percentage of fluorophore-conjugated antibodies were obtained following unsupervised discovery of PBMC populations, gated in reference to an unstained sample. Statistical analysis was carried out using Welch’s t-test to determine significance between values (*p< 0.05; **p< 0.01).
