# Supplementary material for: Immune checkpoint inhibition enhances memory T-cell responses to CEF(T), SARS-CoV-2 spike proteins and ChAdOx1 nCoV-19
Source: Hum Vaccin Immunother. 2026 Oct 6;22(1):2742035. doi: 10.1080/21645515.2026.2742035 (PMC13644633; doi:10.1080/21645515.2026.2742035)
Supplement: Supplementary file 2 [file KHVI_A_2742035_SM9822.docx]

| **Study ID** | **Patient ID** | | **Age** | **Sex** | **Ethnicity** | **Cancer type** | **ICI therapy** |
| --- | --- | --- | --- | --- | --- | --- | --- |
| **HIC-COH-100** | | **Patient 1** | 62 | Male | White European | Non-small cell lung cancer | Atezolizumab  (anti-PD-L1) |
| **HIC-COH-111** | | **Patient 2** | 53 | Male | White European | Head and neck cancer | Pembrolizumab  (anti-PD-1) |
| **HIC-COH-114** | | **Patient 3** | 60 | Male | White European | Renal cancer | Nivolumab  (anti-PD-1) |

**Supplementary Table 1. Demographic and clinical information of patients receiving immunotherapy (n=3)**. Demographic information (sex/age/ethnicity) and clinical information (cancer type and treatment) reported for patients receiving ICI treatment and studied within *in vitro* T-cell assays
